# Supplementary material for: Foxp3+ regulatory T cells maintain the bone marrow microenvironment for B cell lymphopoiesis
Source: Nat Commun. 2017 May 9;8:15068. doi: 10.1038/ncomms15068 (PMC5436085; doi:10.1038/ncomms15068)
Supplement: Supplementary Information — Supplementary Figures [file ncomms15068-s1.pdf]

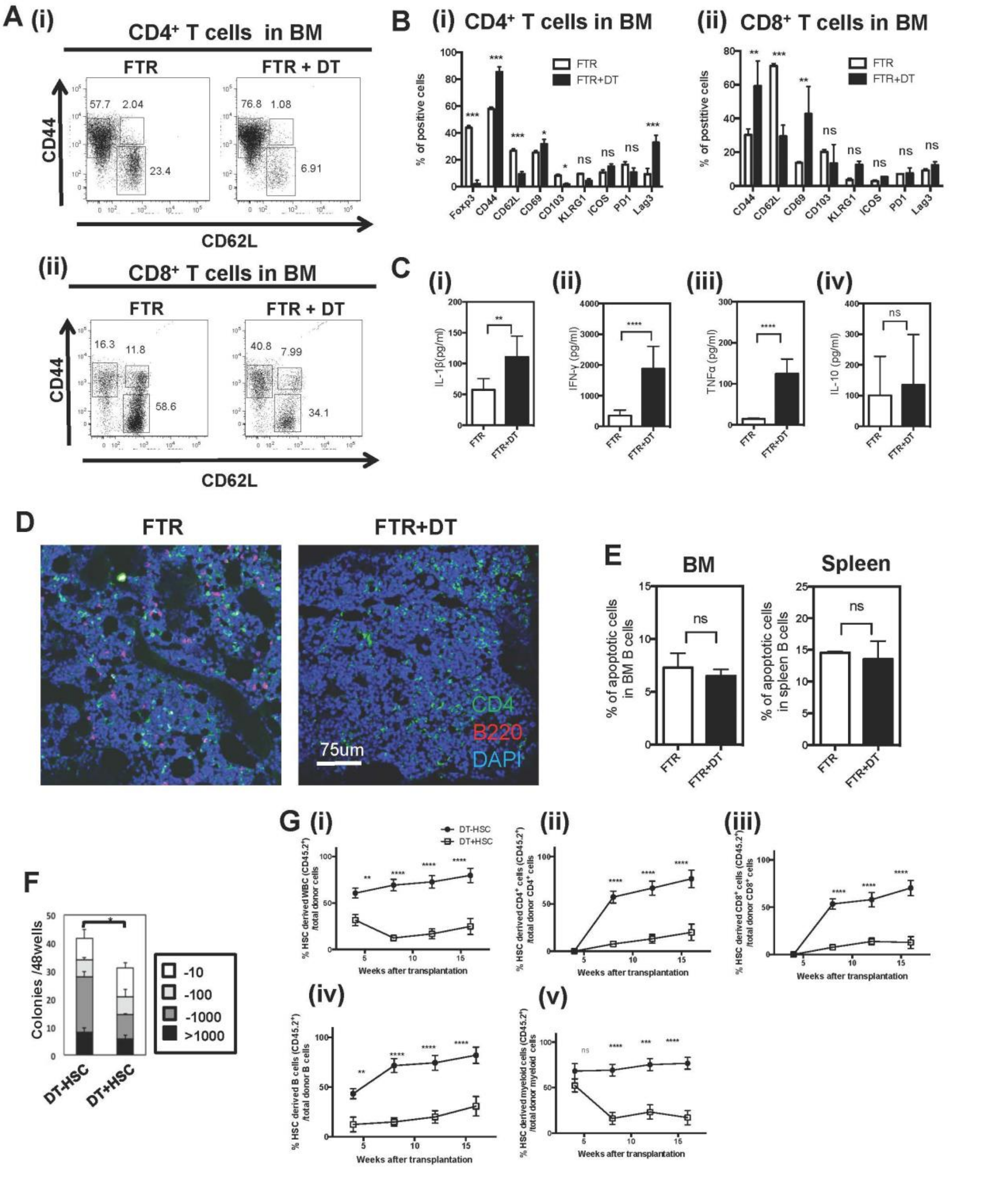

Supplementary Fig 1. Activation of self-reactive BM T cells following Treg depletion.

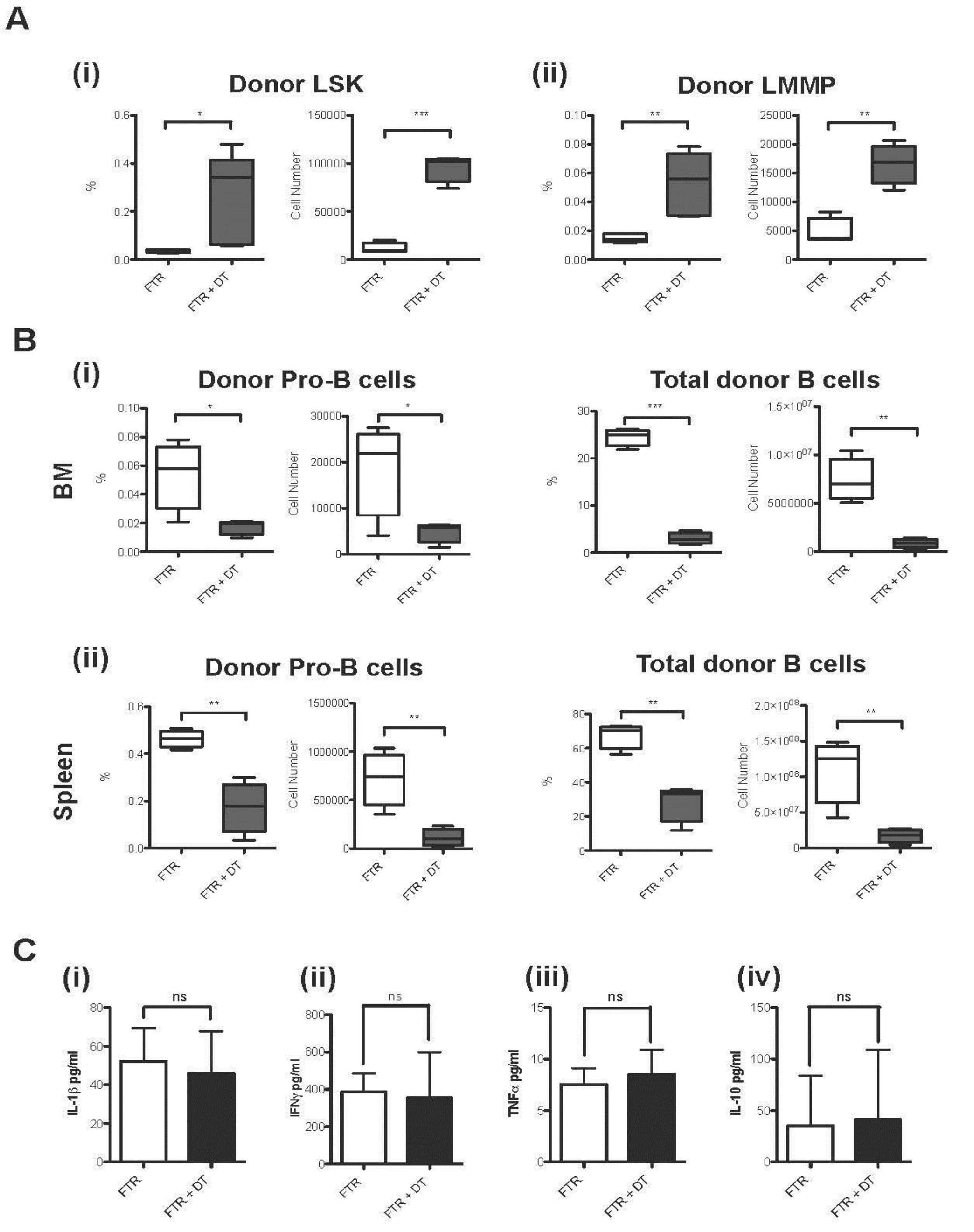

Supplementary Fig 2. Host-Treg depletion induces a block in the early phases of B cell differentiation after syngeneic transplantation

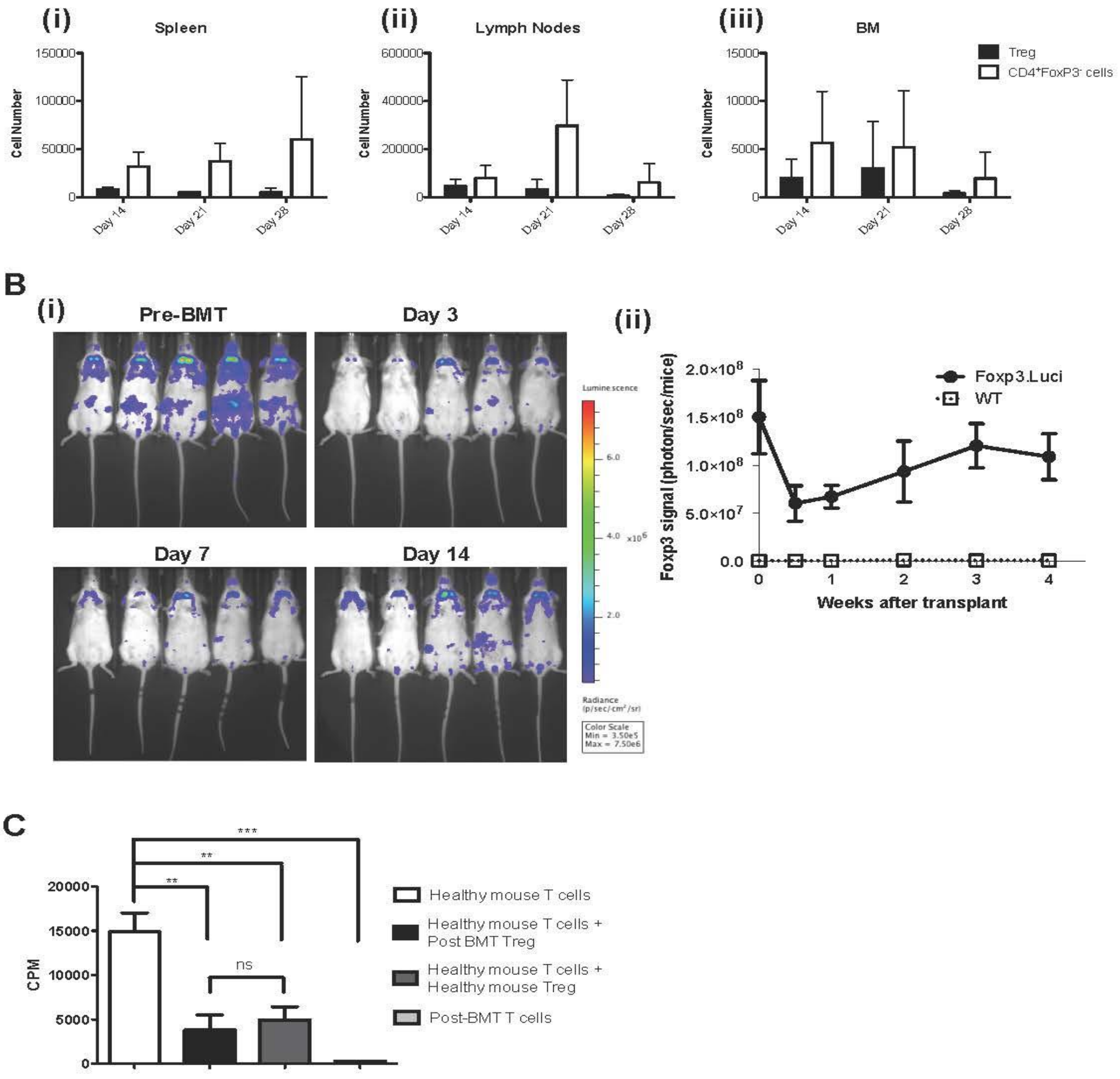

Supplementary Fig 3. Residual host derived Treg after transplantation retain suppressive function.

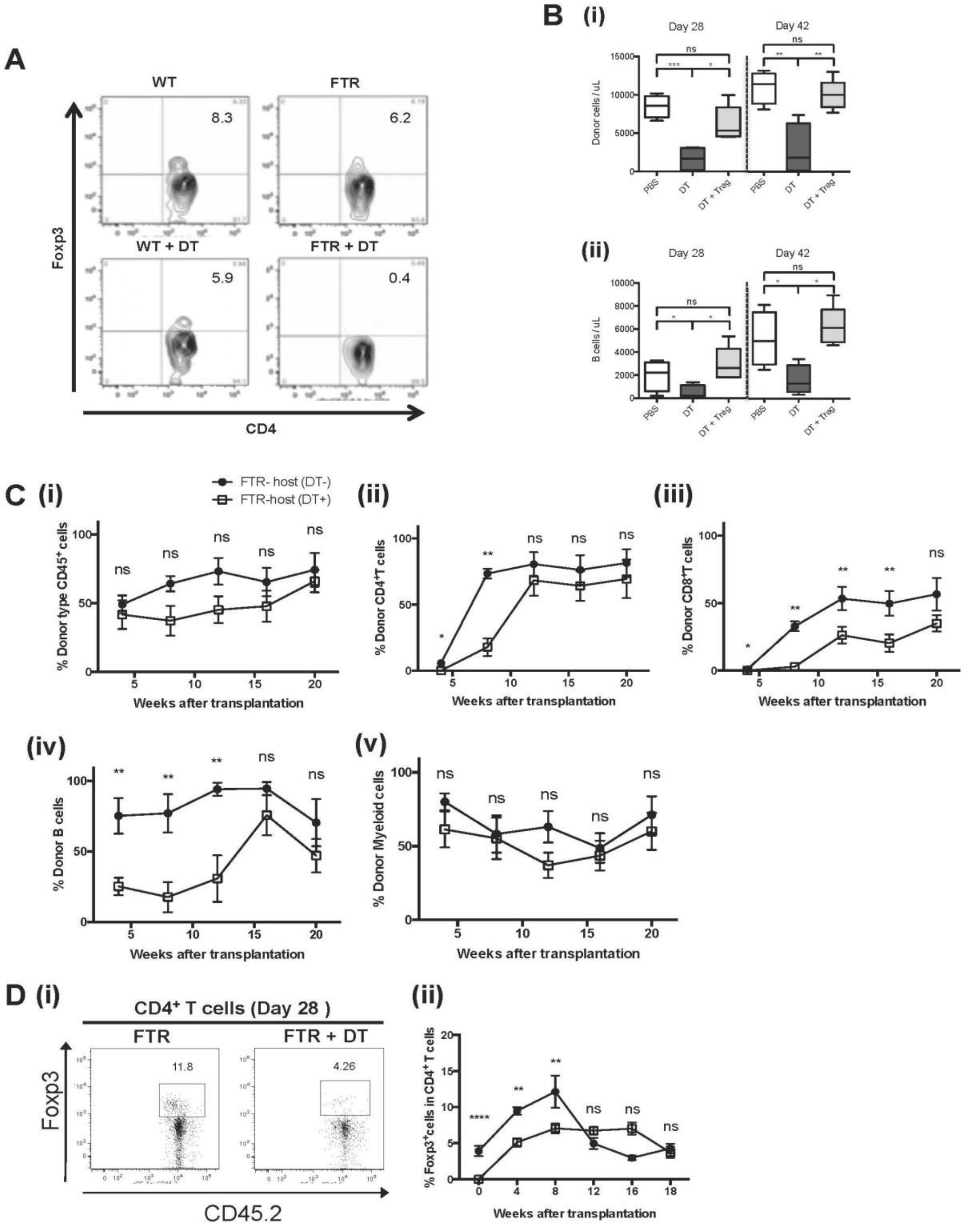

Supplementary Fig 4. Host Treg adoptive transfer rescues donor engraftment and B cell reconstitution in Treg-depleted allogeneic transplanted mice.

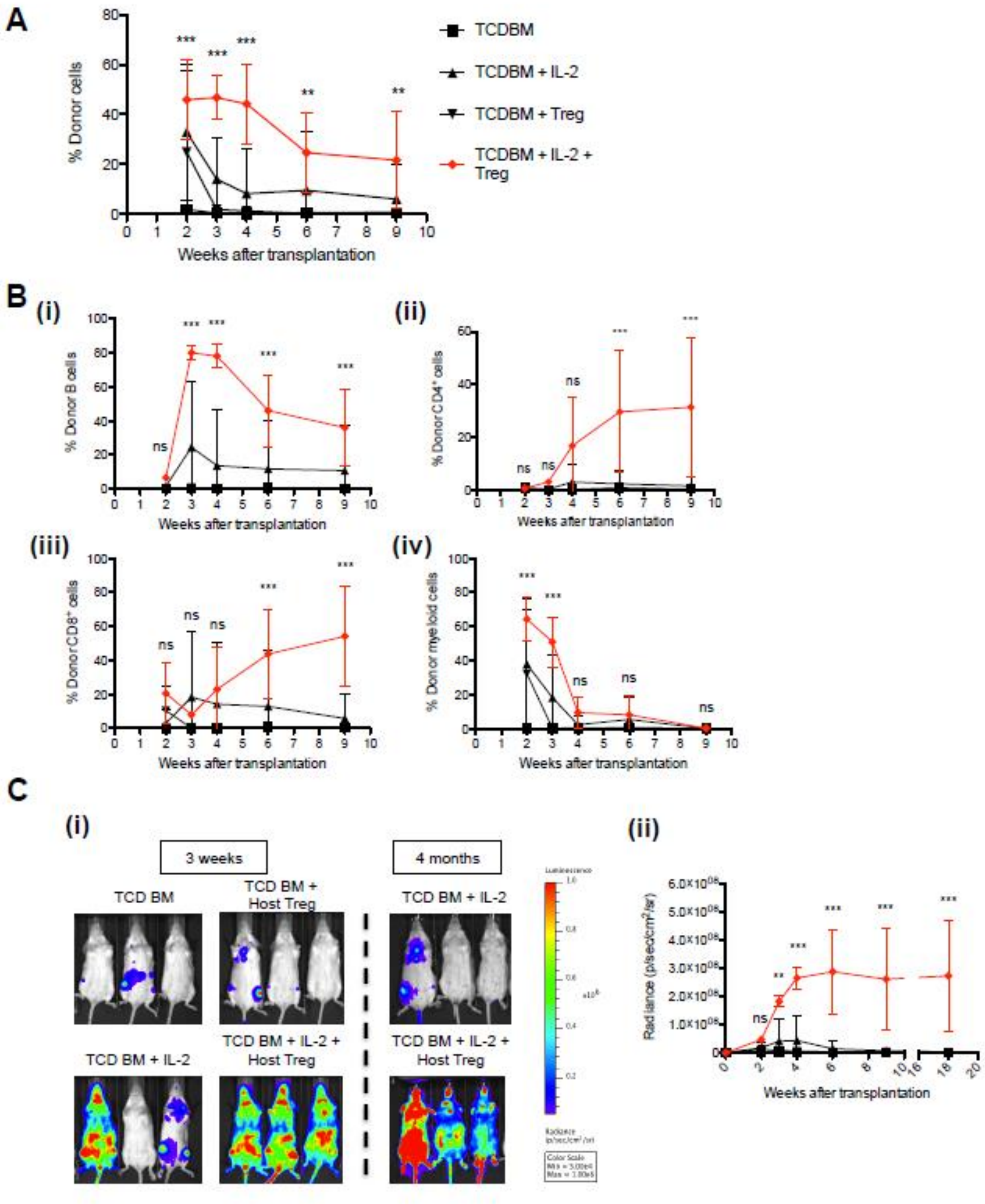

Supplementary Fig 5. In vivo treatment with host Treg and low doses of IL-2 promotes donor engraftment and donor lymphoid reconstitution in sublethally irradiated transplanted immune competent mice

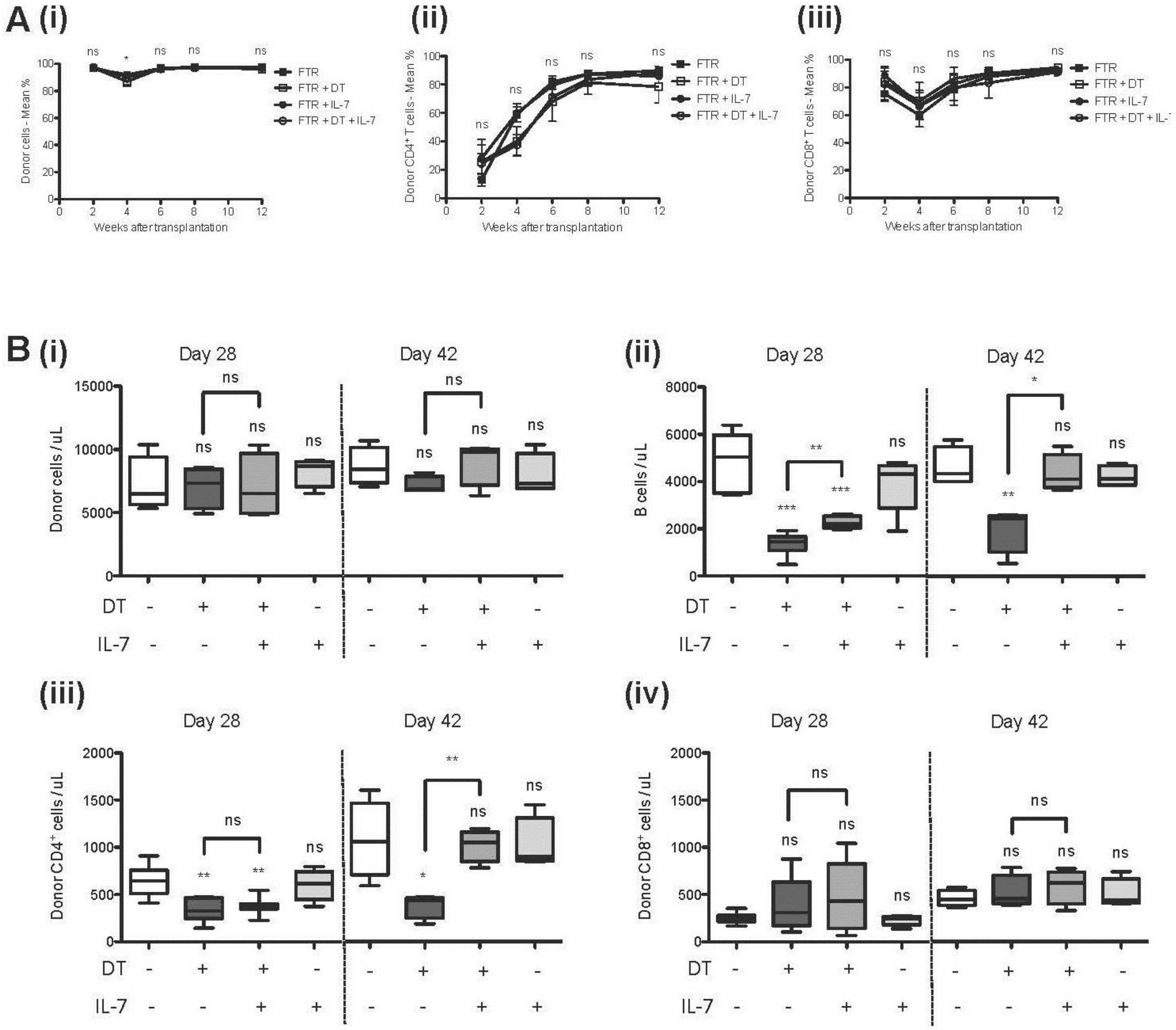

Supplementary Fig 6. IL-7 treatment rescues donor B cell reconstitution without impacting other lymphoid cell lineages in syngeneic transplanted Treg-depleted mice

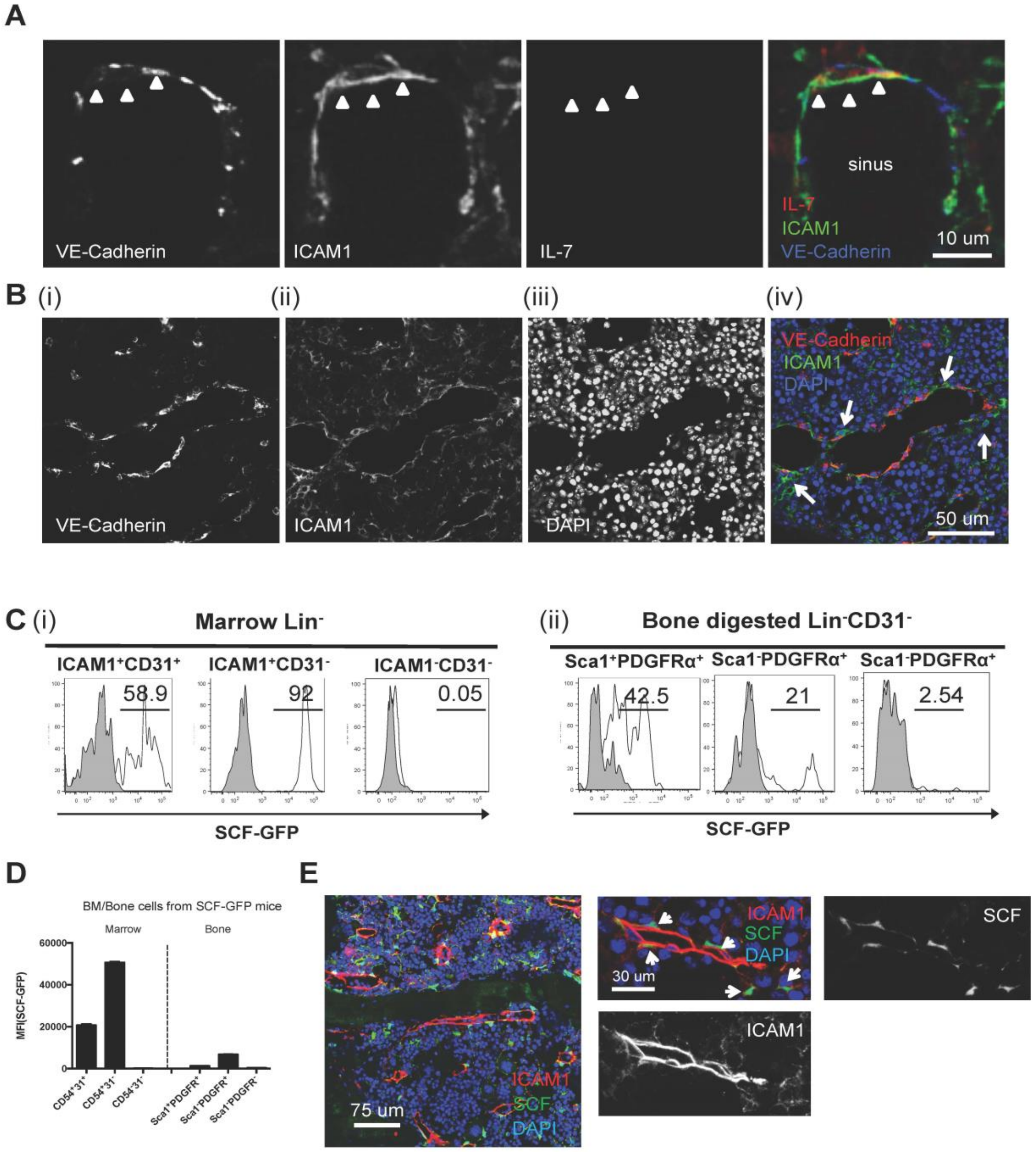

Supplementary Fig 7. Non endothelial ICAM1^{+} cells are perivascular stromal cells and the majority of ICAM1^{+}CD31^{-}CD45^{-}TER119^{-} non hematopoietic cells in BM are SCF-secreting niche cells.
